# Dental Hints

**Published:** 1904-10

**Authors:** 


### DENTAL HINTS.

Oxyphosphate Fillings in the Anterior Teeth of Children.—Dr. Ottolengui considers the practice of placing gutta-percha or oxyphosphate in proximal cavities in the anterior teeth of children reprehensible in ninety per cent. of all cases, as the final filling of gold will be larger than if inserted at the first treatment of the cavity.—*H. E. Eaton, Dominion Dental Journal.*

Plaster Impressions.—When a plaster impression is to be taken of a mouth, especially when some teeth remain in it, and it is free from thick saliva, it should be rinsed with a saponaceous wash to prevent parts of the impression material breaking away and sticking to the dry mucous membrane.

Partial Plate.—In making a partial rubber plate, when the bite is close, solder a flange of platinoid under the heads of the platinum pins, perforate it with holes and let it extend in the body of the plate. If carefully heated "rubber teeth" will not crack when soldered with fine gold solder and the platinoid will not melt under 20 carat fine solder.

Bands in Regulating.—A nickel rolled out to the proper thickness serves the purpose far better than German silver. It is better in color, and remains so; is somewhat stronger, and can be soldered with the greatest ease.—*Brief.*

Concerning Arkansas Stones.—Wash your Arkansas stone with soap and water and never again use oil upon it. While operating keep it on the bracket table and sharpen all cutting instruments, with water only, before using. Water is more cleanly, efficient and presentable than oil. It oxidizes the particles of iron and the oxide aids in producing a keen edge.—*Dr. W. C. Gowan, Review.*

---

Moistening Root Canals Before Filling.—Tyndale's pure oil of eucalyptus is an excellent dressing with which to moisten root canals prior to introducing gutta-percha cones. If also applied after the cone has been placed in the canal, and by employing a blast of warm air the gutta-percha will be easily packed into the canal. Just enough of the oil to moisten the canal is all that is necessary.—*C. R. Taylor, Review.*

---

To Make a Finger-Hold for Broaches.—Ordinary Donaldson broaches may be changed to handy broaches for molars by cutting off half the stem or handle and dropping two or three drops of ordinary sticky wax on the end to form a globule or finger-hold.—*J. C. Montgomery.*

---

Deciduous Teeth with Putrescent Pulps.—Remove all debris as far as possible and insert a small pledget of cotton saturated with Dr. Black's 1, 2, 3; fill cavity with oxyphosphate or with gutta-percha; if with the latter, add to it as it wears down. This will insure the comfort of the little patient and assure mastication.—*Dr. G. E. Adams, International Dental Journal.*

---

Erosion.—In cases of erosion make the fillings concave on the surface to protect margins from acid secretions, which will then accumulate in the saucer-like depression and expend their effect on the filling material.—*Dr. F. W. Stephen, Review.*

---

In approximal cavities of molars and bicuspids there should be a step cut in the occlusal surface and this should be but slightly dovetailed or grooved, as deep undercuts have a tendency to weaken the tooth, and are not necessary for anchorage.—*Dr. H. P. Martin, Register.*

---

To Apply Rubber Dam to Lower Incisor Teeth.—First tie a ligature around the teeth on which you desire to put the dam; then apply the dam and ligate in the usual way. This prevents the stretching of the dam over the ligature from pressure of the lower lip as the dam is held firmly between the two ligatures.—*J. C. Montgomery.*

---

Amalgam Fillings in the Deciduous Molars.—In compound cavities, in cases of near proximity to the pulp, or in extra resistive dentin, carbonize the cavity, varnish, fill half full with cement, and follow with amalgam, pressing firmly to displace the cement, except a mere lining.—*Garrett Newkirk, Dental Review.*

---

Quack Dentists.—It is true beyond question that hundreds of people will and do employ quack dentists who would not think of employing a quack physician. This is true because we have made the purely mechanical side of our work the basis for charge and have given the scientific and professional part free.—*Dr. S. H. Voyless, Era.*

---

Overcome Gagging. — Dipping the plate into strong vinegar before inserting it in the patient's mouth will prevent any ordinary case of gagging. In extreme cases, bathe the roof of the mouth with a menthol solution applied on a pledget of cotton.—*Dr. C. L. Davis, Summary.*

---

Polish for Aluminum. — Procure from a hardware dealer a supply of universal metal polish (Putz Pomade) and apply the smallest amount possible to a thin cotton wheel by means of a piece of wood, upon which a small amount of polish has been placed, and held against the wheel while running the lathe. The polish should be used without water.—*Dr. J. Mills, Review.*

---

Root-Filling Material.—Gutta-percha base-plate, by weight, one-half ounce; saturate solution of thymol in eucalyptus, by measure. Dissolve the gutta-percha (one-half ounce) in chloroform, add the thymol and eucalyptus and mix thoroughly; then allow the choloroform to evaporate. Work into the canal with broach and force to apex with soft rubber and gutta-percha cone. After five years have found distinct odor of thymol and eucalyptus.—*B. L. Cochran, Dental Review.*

---

Power to Work an Atomizer.—The ordinary rubber bulb sold with atomizers is of very little use, as sufficient pressure cannot be obtained to produce the required spray. I use a bicycle foot pump attached to the atomizer with a piece of rubber tubing. This will be found convenient to anyone who has occasion to use an atomizer.—*Dr. Oliver Martin, Review.*

---

Method of Making Surface of Wax Smooth.—A smooth surface on a "waxed up case" can be obtained without spoiling festoons or gum carvings by adopting the following method: Smear the surface of the wax with a pellet of cotton saturated in chloroform. Burn off the chloroform with an alcohol lamp or a small bunsen flame. This will leave a smooth, glossy surface on the wax and will not destroy previously carved gums.—*Dr. H. H. Hancock, Review.*

---

When gold is used for banding a tooth in which there is an amalgam filling, the gold may become permeated with mercury and so weakened that eventually it may break up. A piece of platinum foil may be burnished over that part of the band which requires guarding, and soldered to it before it is bent, and thus obviate the danger.—*A. Drake, in Dental Record.*

---

A Little Point to Remember When Inserting a Mechanical Separator.—Whenever the separating points go higher than they should, crowding uncomfortably on the soft tissues, the screws should be relaxed, the points held in the right position while pieces of gutta-percha are warmed, and crowded under the bows. When these are hard (and the hardening may be hastened with the chip-blower) the upward movement of the points is prevented, and separation is proceeded with as usual.—*Dr. Garrett Newkirk, Dominion Dental Journal.*

---

Reciprocity.—If one may judge from the expressions of opinion recorded in our literature there is an increasing sentiment in favor of the right to practice dentistry anywhere in the Union by those who have once qualified under a proper state law. It is clear that the vital obstacle to be overcome is the lack of uniformity in state dental legislation; states having a high standard refuse to accept the licenses of states having a lower standard, and low standard states decline to accept the licenses of high standard states unless the recognition be reciprocal.—*Dr. E. C. Kirk, Items.*

